# Macroeconomic changes and trends in dental care utilization in Estonia and Lithuania in 2004–2012: a repeated cross-sectional study

**DOI:** 10.1186/s12903-018-0665-5

**Published:** 2018-12-03

**Authors:** Mall Leinsalu, Rainer Reile, Kaire Vals, Janina Petkeviciene, Mare Tekkel, Andrew Stickley

**Affiliations:** 1 Department of Epidemiology and Biostatistics, National Institute for Health Development, Hiiu 42, 11619 Tallinn, Estonia; 2 The Stockholm Center for Health and Social Change (SCOHOST), Södertörn University, 141 89 Huddinge, Sweden; 3 Department of Drug and Infectious Diseases Epidemiology, National Institute for Health Development, Hiiu 42, 11619 Tallinn, Estonia; 4 Faculty of Public Health, Medical Academy, Lithuanian University of Health Sciences, Tilzes 18, LT-47181 Kaunas, Lithuania

**Keywords:** Dental care utilization, Social inequalities, Education, Employment

## Abstract

**Background:**

The aim of this study was to assess trends and inequalities in dental care utilization in Estonia and Lithuania in relation to large-scale macroeconomic changes in 2004–2012.

**Methods:**

Data on 22,784 individuals in the 20–64 age group were retrieved from nationally representative cross-sectional surveys in 2004, 2006, 2008, 2010 and 2012. Age- and sex-standardized prevalence estimates of past 12-month dental visits were calculated for each study year, stratified by gender, age group, ethnicity, educational level and economic activity. Multivariable logistic regression analysis was used to assess the independent effect of study year and socioeconomic status on dental visits.

**Results:**

The age- and sex-standardized prevalence of dental visits in the past 12 months was 46–52% in Estonia and 61–67% in Lithuania. In 2004–2008, the prevalence of dental visits increased by 5.9 percentage points in both countries and fell in 2008–2010 by 3.8 percentage points in Estonia and 4.6 percentage points in Lithuania. In both countries the prevalence of dental care utilization had increased slightly by 2012, although the increase was statistically insignificant. Results from a logistic regression analysis showed that these differences between study years were not explained by differences in socioeconomic status or oral health conditions. Women, the main ethnic group (only in Estonia), and higher educated and employed persons had significantly higher odds of dental visits in both countries, but the odds were lower for 50–64 year olds in Lithuania.

**Conclusions:**

In European Union countries with lower national wealth, the use of dental services is sensitive to macroeconomic changes regardless of the extent of public coverage, at the same time, higher public coverage may not relate to lower inequalities in dental care use.

## Background

Oral health is essential for oral functioning, general health, self-esteem and quality of life. Despite this, an estimated 90% of the world’s population suffer from some form of oral disease during their lifetime [1]. Untreated caries, severe periodontal disease and tooth loss accounted for 16.9 million disability-adjusted life years (DALYs) globally in 2015, a 64% increase from 1990 [2]. This rise is alarming given that oral diseases have been associated with chronic conditions such as cardiovascular disease, chronic respiratory disease and poorer cognitive functioning [3–5]. In addition, dental esthetics are important for physical attractiveness and have been linked to both psychological well-being and better outcomes in the labour market [6, 7].

Socioeconomic factors are associated with an increased risk for oral health problems [8, 9] which may be related to differences in dental care utilization. Wamala et al. [10] showed for example, that in Sweden, access to dental care explained 60% of the socioeconomic differences in oral health, while lifestyle factors explained less than 30%. Provision of dental care services, including their availability and affordability depends on countries’ health care policies and funding systems. In most European Union countries the adult population can obtain some subsidized dental treatment either from the allocation of general taxation funds or through health insurance systems, however, population coverage, as well as the range of subsidized treatments varies widely between countries [11, 12]. Unlike other types of health care services where access to a doctor is ensured at a relatively low or no cost for patients, dental care is only partially covered by public funding with out-of-pocket expenses comprising a significant proportion of total dental expenditure [12, 13]. This means that the use of dental services is heavily dependent on personal economic resources.

The availability and affordability of services are important determinants of dental care utilization and of inequalities in the use of dental care. Previous research has shown a strong association between income, household wealth, educational level and dental care utilization [14–16]. Furthermore, there is evidence that countries with higher public or social insurance funded dental care coverage have lower socioeconomic inequalities in the use of dental services [17, 18]. Macroeconomic conditions can directly affect access to dental care through their impact on job opportunities and household income as well as on health care funding that may alter the availability of public dental services and the extent to which services are subsidized. The onset of the global financial crisis in 2008 had a large impact on national economies all over the world, especially in relation to public budgets [19]. In connection with this, studies have shown that since 2008, unmet dental care needs have increased in many countries in Europe, but disproportionately more among the lowest income groups or unemployed resulting in widening inequalities in dental care utilization [18, 20].

Despite this research, as yet, there have been comparatively few studies that have examined the effects of both economic growth and recession on dental care utilization, especially in the less wealthy European Union countries. This is an important omission when it comes to understanding the relation between economic processes and dental care usage. The Baltic countries experienced huge macroeconomic change in the 2000s, thus offering a natural experiment for exploring its effects on health and health-related behaviours. In Estonia and Lithuania, per capita gross domestic product (GDP) more than doubled between 2004 and 2008 but then both countries experienced a sharp decrease in per capita GDP with about 20% reduction occurring from 2008 to 2009. Although this recession was relatively short-term and per capita GDP had already surpassed the pre-recession level by 2013, it nevertheless remained two (in Estonia) or even more than two times lower (in Lithuania) compared to the average in the European Union in 2012 [21]. Given this, the aim of the current study was to assess the trends and inequalities in the use of dental care services in Estonia and Lithuania in relation to large-scale macroeconomic changes in 2004–2012. We further aimed to examine to what extent differences in dental care utilization between study years and population groups could be accounted for by differences in educational level, employment status and oral health indicators.

## Methods

### Data sources

Nationally representative cross-sectional data for the 2004–2012 period were obtained from five successive surveys of the Health Behaviour among the Adult Population undertaken in Estonia and Lithuania. The surveys used a harmonized methodology and questionnaires and were part of the collaborative Finbalt Health Monitor project, a series of biennial postal surveys focusing on health-related behaviours and outcomes [22]. The surveys were based on random samples of 3000–5000 16–64 year old individuals (depending on the country and study year) derived from national population registries. The overall response rates varied between 59 and 63% in Estonia (*n* = 13,750) and 52–62% in Lithuania (*n* = 9034) (Table 1). Item non-response varied between 0 and 3% and respondents with missing information for any of the study variables were excluded from the analytical sample (471 in Estonia and 307 in Lithuania). For comparability, this study included respondents in the 20–64 age group.

**Table 1:** Characteristics of the surveys and samples, 2004–2012

	Estonia	Lithuania
	*n* (response rate, %)	*n* (response rate, %)
Study year		
2004	2781 (63)	1807 (62)
2006	2585 (59)	1739 (59)
2008	2758 (62)	1731 (61)
2010	2842 (62)	1972 (54)
2012	2784 (62)	1785 (52)
Characteristics of the samples (%)^a^		
	(*n* = 13,750)	(*n* = 9034)
Gender		
Men	41 (43, 42, 42)	41 (43, 42, 40)
Women	59 (57, 58, 58)	59 (57, 58, 60)
Age		
20–34	33 (33, 33, 30)	30 (31, 29, 27)
35–49	33 (33, 34, 32)	37 (38, 39, 37)
50–64	35 (33, 33, 37)	33 (31, 32, 36)
Ethnicity		
Main	70 (68, 69, 72)	87 (86, 87, 87)
Other	31 (32, 31, 28)	13 (14, 12, 13)
Missing	0 (0, 0, 0)	1 (1, 1, 0)
Education		
High	25 (19, 26, 29)	26 (24, 29, 27)
Mid	62 (65, 62, 60)	64 (63, 64, 65)
Low	13 (15, 12, 11)	10 (13, 7, 8)
Missing	0 (1, 1, 0)	0 (0, 0, 1)
Economic activity		
Employed	72 (72, 77, 70)	75 (72, 80, 74)
Non-employed	27 (27, 23, 28)	24 (27, 19, 25)
Missing	1 (1, 1, 2)	1 (1, 1, 2)
Dental visits		
Yes	49 (46, 53, 49)	64 (62, 68, 65)
No	51 (54, 47, 51)	36 (38, 32, 35)
Toothache		
Yes	15 (17, 13, 14)	13 (13, 12, 14)
No	84 (82, 85, 84)	87 (87, 88, 87)
Missing	2 (1, 2, 2)	0 (0, 0, 0)
Missing teeth		
No	22 (18, 25, 23)	19 (16, 21, 20)
1–5	47 (48, 48, 47)	48 (48, 47, 47)
> 6	30 (33, 27, 30)	32 (34, 29, 32)
Missing	1 (1, 1, 0)	2 (2, 3, 1)

^a^Average proportion for 2004–2012 and point estimates for 2004, 2008 and 2012

### Measures

The use of dental care services was measured with the question “How many times did you visit a dentist during the past 12 months?” Respondents were classified as having had at least one visit or not having any visits.

The use of dental care services was assessed in relation to age, gender, ethnicity, educational level, economic activity and oral health. Age was analysed in three groups: 1) 20–34, 2) 35–49, and 3) 50–64 years old. Data on self-reported ethnic identity were categorized into the 1) main ethnic group referring to ethnic Estonians or ethnic Lithanians, and 2) other ethnic groups. Education was measured by the highest level of completed education and was categorized as 1) high, referring to university level education, 2) intermediate, covering upper secondary education, and, 3) low level, referring to the remaining lower levels of education. Economic activity was dichotomized into 1) employed (including self-employed), and 2) non-employed categories, the latter consisting of respondents who were either studying, were homemakers or retired, or who were unemployed. We used two indicators for oral health: toothache in the past 12 months categorized as 1) yes and 2) no toothache, and number of missing teeth where predefined answer categories were aggregated into three groups: 1) none, 2) 1–5, and, 3) 6 or more missing teeth.

### Statistical analysis

In this study, macroeconomic variation was determined by study year: 2004–2008 represented a period of strong economic growth, 2008–2010 was denoted by a period of deep recession, while 2012 represented a partial economic recovery. Age- and sex-standardized prevalence estimates (direct method; “old” European standard population) were calculated for each study year to assess the overall and group specific trends in dental care utilization. Multivariable logistic regression analysis was then used with pooled data to investigate social variation and changes in dental care utilization between study years as compared to in 2008 (the year with the highest per capita GDP) [21] to assess the impact of both economic growth and recession on dental visits in the two countries. Three models were analysed. Model 1 was adjusted for age, gender and ethnicity; subsequent models were additionally adjusted for educational level and economic activity (Model 2), and for toothache in the past 12 months and number of missing teeth (Model 3). The results from the logistic regression analyses are presented as odds ratios (ORs) with 95% confidence intervals (CIs). The statistical analyses were conducted using SPSS Statistics for Windows, version 24.0 (IBM Corp. 2016).

## Results

The sample characteristics are presented in Table 1. In both countries more women than men participated in the surveys. Lithuania had more middle-aged respondents, whereas Estonia had a considerably larger proportion of other ethnic groups. Nearly half of the respondents reported a dental visit in the past 12 months in Estonia whereas nearly two-thirds did so in Lithuania. Almost one-third of the respondents had 6 or more teeth missing in both countries including 3% of respondents who were edentulous.

Age- and sex-standardized prevalence estimates for past 12-month dental visits are presented in Table 2. The overall prevalence was considerably lower in Estonia (46–52%) as compared to Lithuania (61–67%) in all study years. In 2004–2008, the prevalence of dental visits increased by 5.9 percentage points in both countries and fell in 2008–2010 by 3.8 percentage points in Estonia and 4.6 percentage points in Lithuania. Except for the lowest educated in Lithuania, dental care utilization increased in all population subgroups in both countries in 2004–2008. The increase was statistically significant for women, 35–64 year olds, ethnic Estonians, the mid-educated group, and for both employed and non-employed persons in Estonia, and for men, ethnic Lithuanians, and employed persons in Lithuania. In 2008–2010, the prevalence of dental visits fell in all population subgroups except for the lowest educated in Lithuania where it remained the same. The decline was statistically significant in women, in the 35–49 age group and in the mid-educated group in Estonia. By 2012, the prevalence of dental care utilization had increased in all groups except for the lowest educated in Lithuania, and the other ethnic group, the mid-educated, and those aged 20–34 and 50–64 in Estonia.

**Table 2:** Age- and sex-standardized prevalence (%) with 95% confidence intervals (CI) for dental visits in the past 12 months, 2004–2012

	Age- and sex-standardized prevalence (%) with 95% CI					Change^a^	
	2004	2006	2008	2010	2012	2004/2008	2008/2010
Estonia							
Total	45.6 (43.7–47.4)	50.5 (48.5–52.5)	51.5 (49.6–53.4)	47.7 (45.8–49.5)	48.8 (46.9–50.7)	** 5.9**	**−3.8**
Gender							
Men	36.7 (33.9–39.4)	42.7 (39.6–45.9)	41.7 (38.8–44.6)	40.1 (37.2–42.9)	42.0 (39.1–44.9)	5.0	−1.6
Women	54.4 (52.0–56.9)	58.3 (55.8–60.8)	61.3 (58.8–63.7)	55.3 (52.9–57.7)	55.6 (53.1–58.1)	** 6.9**	**−6.0**
Age							
20–34	48.3 (45.2–51.5)	54.3 (50.9–57.7)	52.1 (48.8–55.4)	51.1 (47.9–54.4)	50.1 (46.6–53.5)	3.8	−1.0
35–49	48.9 (45.6–52.1)	52.8 (49.3–56.4)	55.8 (52.6–59.1)	46.0 (42.7–49.3)	52.3 (48.9–55.7)	** 6.9**	**−9.8**
50–64	38.4 (35.2–41.7)	43.3 (39.8–46.7)	45.7 (42.4–49.0)	45.6 (42.4–48.8)	43.3 (40.2–46.4)	** 7.3**	−0.1
Ethnicity							
Main	46.8 (44.5–49.0)	51.5 (49.1–53.9)	52.5 (50.3–54.8)	48.1 (45.8–50.2)	49.8 (47.5–52.0)	** 5.7**	−4.4
Other	43.5 (40.1–46.8)	47.7 (44.0–51.3)	48.5 (45.1–52.0)	46.2 (42.7–49.7)	45.5 (41.8–49.1)	5.0	−2.3
Education							
High	59.4 (54.9–64.0)	61.2 (57.0–65.4)	60.0 (55.8–64.2)	57.1 (53.2–61.1)	61.8 (58.0–65.6)	0.6	−2.9
Mid	44.8 (42.5–47.1)	48.5 (46.0–51.0)	50.9 (48.5–53.3)	46.0 (43.6–48.4)	45.8 (43.2–48.3)	** 6.1**	**−4.9**
Low	31.1 (25.4–36.8)	33.8 (28.0–39.5)	32.7 (27.0–38.5)	28.7 (23.0–34.3)	33.1 (27.0–39.3)	1.6	−4.0
Economic activity							
Employed	47.9 (45.7–50.1)	51.8 (49.5–54.1)	52.3 (50.1–54.4)	49.5 (47.2–51.8)	50.7 (48.4–53.0)	** 4.4**	−2.8
Non-employed	37.7 (33.9–41.4)	42.9 (37.9–47.9)	46.7 (41.6–51.9)	42.0 (38.5–45.5)	42.3 (38.0–46.7)	** 9.0**	−4.7
Lithuania							
Total	60.9 (58.6–63.1)	62.9 (60.6–65.2)	66.8 (64.5–69.1)	62.2 (59.9–64.4)	64.8 (62.4–67.1)	** 5.9**	**−4.6**
Gender							
Men	51.3 (47.7–54.9)	53.4 (49.7–57.1)	59.5 (55.8–63.3)	54.0 (50.3–57.7)	55.9 (52.1–59.6)	** 8.2**	−5.5
Women	70.4 (67.6–73.2)	72.4 (69.6–75.1)	74.1 (71.3–76.8)	70.4 (67.8–73.0)	73.7 (70.9–76.4)	3.7	−3.7
Age							
20–34	63.8 (59.8–67.8)	69.8 (65.8–73.9)	67.4 (63.1–71.7)	64.0 (59.9–68.1)	67.4 (63.1–71.8)	3.6	−3.4
35–49	64.4 (60.8–68.1)	64.2 (60.4–67.9)	68.0 (64.3–71.6)	63.0 (59.4–66.7)	66.0 (62.2–69.8)	3.6	−5.0
50–64	53.3 (49.1–57.5)	53.3 (49.1–57.5)	64.7 (60.5–68.9)	59.0 (55.0–63.0)	60.1 (56.3–64.0)	11.4	−5.7
Ethnicity							
Main	60.5 (58.1–63.0)	62.8 (60.3–65.2)	66.9 (64.4–69.4)	62.8 (60.4–65.2)	65.0 (62.5–67.4)	** 6.4**	−4.1
Other	62.1 (55.8–68.4)	65.9 (60.1–71.7)	66.6 (60.8–72.5)	57.4 (49.9–64.9)	63.7 (56.9–70.4)	4.5	−9.2
Education							
High	69.2 (64.5–74.0)	73.3 (68.8–77.8)	74.0 (69.7–78.3)	72.8 (68.5–77.0)	78.9 (74.8–83.0)	4.8	−1.2
Mid	60.3 (57.4–63.2)	62.6 (59.7–65.5)	65.3 (62.0–68.6)	59.6 (56.6–62.6)	61.4 (58.3–64.4)	5.0	−5.7
Low	54.8 (47.0–62.7)	47.6 (40.9–54.3)	48.3 (40.4–56.3)	48.3 (41.0–55.6)	41.8 (32.9–50.6)	−6.5	0.0
Economic activity							
Employed	61.7 (59.0–64.4)	64.8 (62.1–67.4)	67.8 (65.2–70.4)	64.3 (61.6–67.0)	66.6 (63.8–69.3)	** 6.1**	−3.5
Non-employed	57.6 (52.3–63.0)	52.6 (46.6–58.6)	60.5 (52.9–68.2)	56.5 (51.1–61.9)	56.7 (51.3–62.1)	2.9	−4.0

^a^Statistically significant changes (*p* < 0.05) are marked in bold font

Results from a multivariable logistic regression analysis (Table 3) showed that after adjustment for age, gender and ethnicity (Model 1), the odds of visiting a dentist were statistically significantly lower in 2004 as compared to 2008 in Estonia, and in 2004 and 2006 as compared to in 2008 in Lithuania. After further adjustment for educational level and economic activity (Model 2), the odds were slightly attenuated and also became statistically insignificant in 2006 for Lithuania. Adjustment for oral health indicators (Model 3) did not have any additional effect on yearly change. As compared to in 2008, the odds of visiting a dentist were statistically significantly lower in 2010 and 2012 in Estonia, and in 2010 in Lithuania in all models with almost no effect observed after adjustment for socioeconomic and oral health indicators. In both countries, women, higher educated and employed persons, and in Estonia also ethnic Estonians had statistically significantly higher odds for past 12-month dental visits in the fully adjusted model. Estonia had a somewhat deeper educational gradient than Lithuania, whereas the opposite was true for economic activity. Although the oldest age group had lower odds for dental visits in models adjusted for demographic and socioeconomic variables, further adjustment for toothache and missing teeth attenuated the association to a non-significant level in Estonia. Having no toothache or 6 or more missing teeth were associated with lower odds for dental visits in both countries.

**Table 3:** Association of study years and socioeconomic variables with dental visits, 2004–2012. Odds ratios (OR) with 95% confidence intervals (CI)

	Estonia			Lithuania		
	Model 1	Model 2	Model 3	Model 1	Model 2	Model 3
	OR (95% CI)	OR (95% CI)	OR (95% CI)	OR (95% CI)	OR (95% CI)	OR (95% CI)
Year						
2004	0.77 (0.69–0.86)	0.81 (0.73–0.91)	0.80 (0.72–0.90)	0.77 (0.67–0.89)	0.83 (0.72–0.96)	0.83 (0.72–0.96)
2006	0.94 (0.84–1.05)	0.96 (0.86–1.07)	0.96 (0.86–1.08)	0.84 (0.73–0.98)	0.89 (0.77–1.04)	0.90 (0.77–1.04)
2008	1	1	1	1	1	1
2010	0.85 (0.76–0.94)	0.85 (0.76–0.94)	0.84 (0.75–0.93)	0.82 (0.71–0.94)	0.85 (0.74–0.99)	0.85 (0.74–0.99)
2012	0.87 (0.78–0.98)	0.86 (0.77–0.96)	0.85 (0.76–0.95)	0.92 (0.80–1.07)	0.95 (0.82–1.10)	0.94 (0.81–1.09)
Gender						
Men	1	1	1	1	1	1
Women	1.96 (1.83–3.10)	1.83 (1.70–1.97)	1.86 (1.73–2.00)	2.17 (1.99–2.38)	2.10 (1.92–2.31)	2.17 (1.97–2.38)
Age						
20–34	1	1	1	1	1	1
35–49	1.00 (0.92–1.09)	0.93 (0.85–1.02)	1.02 (0.92–1.12)	0.94 (0.84–1.05)	0.87 (0.77–0.97)	0.93 (0.82–1.06)
50–64	0.73 (0.67–0.79)	0.75 (0.69–0.82)	0.94 (0.85–1.05)	0.70 (0.63–0.79)	0.72 (0.64–0.81)	0.86 (0.75–0.99)
Ethnicity						
Main	1	1	1	1	1	1
Other	0.88 (0.82–0.95)	0.88 (0.81–0.95)	0.85 (0.79–0.92)	0.94 (0.82–1.08)	0.96 (0.84–1.10)	0.95 (0.83–1.09)
Education						
High		1	1		1	1
Mid		0.59 (0.54–0.64)	0.60 (0.55–0.65)		0.61 (0.54–0.68)	0.62 (0.55–0.69)
Low		0.33 (0.29–0.37)	0.32 (0.28–0.37)		0.40 (0.34–0.47)	0.40 (0.34–0.48)
Economic activity						
Employed		1	1		1	1
Non-employed		0.84 (0.78–0.91)	0.86 (0.79–0.93)		0.74 (0.67–0.83)	0.75 (0.67–0.84)
Toothache						
Yes			1			1
No			0.51 (0.46–0.57)			0.53 (0.46–0.62)
Missing teeth						
No			1			1
1–5			1.16 (1.05–1.28)			1.15 (0.99–1.32)
> 6			0.71 (0.62–0.80)			0.76 (0.65–0.90)

Model 1 – Adjusted for age, gender and ethnicity

Model 2 – Adjusted for age, gender, ethnicity, education and economic activity

Model 3 – Adjusted for age, gender, ethnicity, education, economic activity, toothache in the past 12 months and number of missing teeth

## Discussion

This study examined the use of dental care services in two Baltic countries in a period of profound macroeconomic change. Results showed that there was a statistically significant increase in dental visits in Estonia and Lithuania in 2004–2008 followed by a significant decline in 2010. The differences between study years remained significant after controlling for socioeconomic and oral health indicators. In Estonia, the statistically significant changes covered a broader spectrum of population subgroups compared to in Lithuania. A strong positive socioeconomic gradient in dental visits was found in both countries.

Before discussing this study’s findings, several limitations should be mentioned. First, response rates were relatively low across surveys with younger persons, men and those living in urban areas being less likely to respond. An earlier sensitivity analysis conducted to assess the possible impact of non-response bias on outcome measures using post-stratification weighting showed that differences between the weighted and unweighted prevalence estimates for dental visits were minimal [23]. As respondents generally tend to have a better socioeconomic position compared to non-responders this may have resulted in some overestimation of the prevalence of dental visits although evidence suggests that the effect on associations remains marginal [24, 25]. Thus, it seems unlikely that non-response has affected our results or conclusions in any significant way. Second, we were limited to using repeated cross-sectional data that did not allow us to track individuals over time. Third, we could not distinguish between preventive and curative dental care, although results may differ across both types of care with preventive care being associated with larger inequalities [15]. Finally, although we studied changes over time, macroeconomic measures were not directly included in the analysis. Even though the longer time period covered in this study affords us greater certainty when attributing observed changes in dental visits to macroeconomic changes, we acknowledge, that this presents only one possible explanation. Further research is needed therefore to determine the contribution of other contextual characteristics to yearly variations in dental care utilization in this period.

The prevalence of dental visits was 20–25% lower in Estonia as compared to in Lithuania across the study years. Availability of services is unlikely to explain such a persistent difference over time as in Estonia the per capita number of dentists was higher than in Lithuania, although there has been a stronger growth in numbers in Lithuania in more recent years. In 2012, there were 9.0 and 8.9 practicing dentists per 10,000 inhabitants respectively in Estonia and Lithuania [26]. The high percentage of respondents who had 6 or more teeth missing is alarming, although it was similar in both countries and differences in oral health cannot thus explain the gap in dental visits between the two countries. In Estonia, the insured adult population is entitled to compensation with a ceiling corresponding to one preventive visit annually with higher reimbursement for some vulnerable groups. These cash benefits were stopped in 2009 as part of an austerity package that was implemented [27]. In Lithuania, the cost of dental services provided in public facilities or by private dentists contracted with the National Health Insurance Fund is partly covered by the Fund and partly (for dental materials) by patients themselves [28, 29]. Dental care expenditure as a proportion of total out-of-pocket payments varied between 19 and 27% in 2000–2012 in Estonia [30], whereas in Lithuania the average was about 10% in 2000–2008 [28]. In the Eurobarometer Oral Health Survey 2009, 29% of Estonians reported that they did not visit a dentist in the past 2 years because it was too expensive, compared to 22% of Lithuanians [31]. It is thus more likely that differences in the level of subsidized services for the adult population may explain much of the difference in dental care visits observed between the countries, even though Estonia’s per capita GDP in 2012 was 21% higher than in Lithuania [21]. This accords with earlier research showing a higher prevalence of dental visits in European countries with greater dental care coverage [17].

The use of dental care services increased in Estonia and Lithuania until 2008. As major oral health care reforms were implemented in these countries before our study period [28], the increased utilization rates are likely to reflect the strong increase in per capita GDP [21]. After the onset of the global economic crisis, the use of dental services declined by about 7% in both countries in 2010, although the negative trend was temporary and the prevalence of dental care utilization had increased slightly by 2012. The cash benefits for dental services that were halted in 2009 in Estonia [27] were not restored until recently which may explain the somewhat slower recovery in dental visits in Estonia. Similar to our results, foregone dental care increased during the recent economic recession in Spain and the United States [20, 32]. A more recent comparative study reported an increase in foregone dental care in 19 out of 23 European countries in 2008–2013 as a result of the economic recession [18]. Out-of-pocket payments comprise a significant proportion of dental care costs [12, 30], which creates a heavier burden on people with fewer monetary resources during economic contractions. This argument is supported by studies that showed a greater increase in unmet dental care needs among groups with a low income, the unemployed and the least educated, during the recent recession [18, 20, 32]. In contrast to these studies, Huang et al. [33] reported a stronger association between unemployment and unmet dental care needs among the unemployed from middle- and high-income families in the United States during the recession which was explained by the differential impact of job loss on meeting higher- and lower-income families’ health care needs. Another study from the United States showed a decline in preventive dental care utilization among dentally insured persons during periods of high unemployment [34]. Having a check-up as a reason for the last dental visit was more frequently reported by women and higher socioeconomic groups in the Eurobarometer Survey 2009, and it was generally low in Estonia (34%) and Lithuania (21%) [31]. Differences in consumption patterns between socioeconomic groups may explain why this study did not find a systematically greater decline in dental visits among lower socioeconomic groups during the recession, and may also explain the stronger decline in dental visits in 2008–2010 among women in Estonia. At the same time, the economic growth in the Baltic countries in the mid-2000s was mostly driven by a boom in mortgage credit, which led to an enormous growth in household debt, especially in higher income households [35]. The higher loan burden may explain why higher socioeconomic groups, although less exposed to unemployment, had to cut their dental care costs when the recession occurred. It is also possible that a higher level of indebtedness among the middle-aged could similarly account for the stronger decline in dental visits they experienced in 2010 as observed in Estonia.

A strong positive association was found between educational level, employment status and the use of dental care services in both countries which is in line with the findings from other studies showing lower utilization rates among less advantaged socioeconomic groups [14, 15, 17]. Although Lithuania had higher dental care coverage, the magnitude of inequalities in dental care use was very similar in both countries. Compared to other European countries where higher public coverage has been linked to lower inequalities in dental care utilization [17], in Lithuania, where per capita GDP is still relatively low [21], the partial coverage of services may not be sufficient to remove barriers to using dental care services among lower socioeconomic groups, although it may have increased overall levels of dental care utilization. The observed ethnic differences in dental visits in Estonia were not explained by differences in socioeconomic status or by oral health conditions, however, we were not able to control for differences in income. Women had higher odds of dental visits compared to men in both countries which might be explained by different norms in help seeking behaviours in men and women [36]. The odds of visiting a dentist were lowest for the oldest age group although oral health problems increase with age. Adjustment for oral health indicators (including edentulism) only partly explained why older people had less dental visits in this study, and thus the high cost of services is a more likely explanation. Evidence from Finland showed that oral health care reform in 2001–2002 that removed barriers preventing the adult population from accessing public and subsidized private basic dental services, subsequently increased the use of services in the older population [37].

## Conclusions

Macroeconomic changes in 2004–2012 had a similar impact on the use of dental care services in the two Baltic countries despite differences in the public coverage of dental care, although the overall prevalence of dental visits was considerably lower in Estonia where public coverage for the adult population was low. Higher educated and employed individuals had significantly higher odds for dental care visits, but socioeconomic inequalities were not smaller in Lithuania. Overall, the findings of this study suggest that in European Union countries with lower national wealth the use of dental services is sensitive to macroeconomic changes regardless of the extent of public coverage, but at the same time, higher public coverage may not relate to lower inequalities in dental care use.
